# Preparation and Curing Mechanism of Modified Corn Straw by 3-Glycidyl Ether Oxypropyl Trimethoxysilane/Epoxy Resin Composites

**DOI:** 10.3390/polym14235233

**Published:** 2022-12-01

**Authors:** Chunhua Lou, Siyu Jiang, Yongli Zhou, Xiaohua Gu, Yong Zhang, Xianzhi Kong

**Affiliations:** 1 School of Chemistry and Chemical Engineering, Qiqihar University, Qiqihar 161006, China; 2 Guilin Institute of Aerospace Technology, School of Energy and Building Environment, Guilin 541004, China; 3 Zibo Luray Fine Chemical Co., Ltd., Zibo 255000, China; 4 Institute of Petrochemistry, Heilongjiang Academy of Sciences, Harbin 150040, China

**Keywords:** epoxy resin, corn straw, composites, preparation, curing mechanism

## Abstract

A modified corn straw (CS)/epoxy resin (EP) composite was prepared using bisphenol A EP (i.e., E-51) as matrix, 2-methylimidazole as curing agent, and CS modified by 3-glycidyl ether oxypropyl trimethoxysilane (KHCS) as filler. Its chemical structure was characterized by Fourier transform infrared spectroscopy (FTIR). The dynamic thermodynamic properties, mechanical properties, flame retardant property, and fracture morphology were studied using dynamic mechanical analysis (DMA), a universal testing machine, a micro combustion calorimeter, and a scanning electron microscope (SEM), respectively. The effects of different contents of KHCS on various properties were discussed. The experimental result showed that the CS was bonded toKH560 by a covalent bond. The impact strength, tensile strength, and flexural strength of the composites were all improved compared with those of pure EP. When the content of KHCS was 15 wt%, the maximum impact strength of the composites was 3.31 kJ/m^2^, which was 1.43 times that of the pure EP. The p HRR and THR of MCSEC-20 were 512.44 W/g and 25.03 kJ/g, respectively, which were 40.71% and 27.76% lower than those of pure EP, when the content of KHCS was 20 wt%. Moreover, the mechanism of the curing composites was investigated.

## 1. Introduction

Epoxy resin (EP) is one of the important thermosetting resins and widely used matrix resins over polymeric materials [1,2]. It has excellent adhesion, wear resistance, mechanical properties, electrical insulation, chemical stability, and high and low temperature resistance, and it has been extensively adopted in adhesives, electronic instruments, aerospace, mechanical engineering, electronic engineering insulation materials, and advanced composites [3,4,5,6,7,8]. However, due to the difficulty in moving the main chain segment resulting from the high cross-linking density, EP has many defects, such as high brittleness, poor flexibility, and weak impact resistance, which greatly limit its application and development in high technology fields [9,10,11,12].

The traditional reinforcement of EP matrix composites comprise carbon fiber, glass fiber, and inorganic powder materials, which often encounter problems, such as resource shortage, difficulty in recycling, and environmental pollution [13]. Different from traditional inorganic and synthetic fibers, natural plant fiber is a kind of fiber material that can be directly obtained from nature. It is not only rich in source, renewable, cheap, and degradable, but also has a higher tensile strength and modulus than traditional inorganic and synthetic fibers [14,15]. Therefore, the natural plant fiber as the reinforcement of EP matrix composites is a new way to modify EP and has become one of the hot spots in the field of epoxy resin reinforcement and toughening [16,17].

China is a traditional agricultural country, and its annual output of crop straw, a kind of agricultural waste, is the highest in the world. At present, such crop straw is mainly used as fuel, animal feed, and industrial energy. However, the material consumption is very small. Most of the agricultural waste straw is directly burned on the spot, which will cause not only substantial waste of this renewable resource but also serious pollution to the ecological environment because of straw burning, which does not accord with the national sustainable development strategy. Therefore, recycling straw resources and improving their actual utilization rate are of great significance to resource conservation and environmental protection. So far, many researchers have modified polymer resin with cellulose and lignin extracted from straw as reinforcement filler. Yin et al. [18] mixed a high content of corn straw (CS) enzymatic hydrolyzed lignin with EP (E51) and the E51/polyamide system to improve the fracture toughness of composites. Zhang et al. [19] prepared a CS cellulose/EP composite with polyisocyanate as a compatibilizer and biuret-modified EP as adhesive to improve the mechanical properties and water resistance of the composite. Nevertheless, the extraction process of cellulose and lignin is complex, and the straw utilization rate is low. To make straw be used more effectively, this study directly used CS to modify EP. Given that cellulose is the main composition of CS, a large number of hydroxyl groups are on the molecular chain, which makes a strong hydrogen bonding interaction among the cellulose chains. Consequently, the interfacial performance between the cellulose molecules and the EP is so poor that EP matrix composites would have some defects, such as fiber peeling, porosity, and easy degradation [16,17,20,21,22,23,24,25,26]. To solve these disadvantages, straw fiber is often treated by physical or chemical modification to improve the interface compatibility between straw fiber and EP matrix [27]. Therefore, in this study, EP matrix composites were prepared using a silane coupling agent as the surface treating agent of CS. Their mechanical performance and thermal performance were investigated, which would provide an experimental basis and a new research approach for the development of sustainable and high-performance composite materials.

## 2. Experimental Section

### 2.1. Materials

Epoxy resin (E-51) of industrial grade (CYD-218), was purchased from China Baling Branch of Sinopec; corn straw (CS) was obtained from the suburb of Qiqihar City, Heilongjiang Province; 2-methylimidazole (2-MI) and acetone of analytical purity were purchased from China Tianjin Kermel chemical reagent development center; 3-glycidyl ether oxypropyl trimethoxysilane (KH560) of excellent grade (99%), was purchased from China Shandong Usolf Chemical Technology Co., Ltd.; and acetic acid of analytical purity was purchased from China Shanghai Aladdin Chemical Reagent Co., Ltd.

### 2.2. Experimental Instruments

The instruments used in this experiment are as follows: WSM-20KN electronic universal testing machine, Intelligent Instrument Equipment Co., Ltd. In China Changchun; GT-7045-HMH impact testing machine, Gotech Testing Machines Inc., China; S-3400 scanning electron microscope, Hitachi High Tech International Trade Co., Ltd., Japan; Frontier online Fourier transform infrared optical fiber detection system, Perkin Elmer instrument Co., Ltd., USA; 204 F1 differential scanning calorimeter, Netzsch Scientific Instruments Trading Co., Ltd., Germany; TGA5500 thermogravimetric analyzer, Waters Science and Technology Co., Ltd., USA; Q800 thermomechanical analyzer, TA Instruments.

### 2.3. Preparation of Samples

#### 2.3.1. Surface Treatment of CS Particles with KH560

First, the crushed CS was pretreated with an acetic acid aqueous solution (pH = 4.5). Second, 2 wt% KH560 was added in a 500 mL three-necked flask, in which a certain proportion of an ethanol aqueous solution was initially added. After the mixture was stirred evenly, the pretreated CS was added. The surface treatment reaction was then stirred in a water bath at 80 °C for 6 h. Next, the product, KHCS, was filtered under reduced pressure after washing with twice with ethanol and three times with distilled water and dried at 120 °C for 48 h. Finally, different sizes of KHCS were obtained by sieving.

#### 2.3.2. Preparation of Modified CS/E51 Composites (MCSECs)

KHCS was mixed with EP in the proportions of 0 wt%, 5 wt%, 10 wt%, 15 wt%, 20 wt%, and 25 wt% in a plastic cup and then placed in an oven for preheating at 60 °C for 20 min. Next, 4 wt% 2-MIwas added into the six plastic cups, which were stirred evenly, and then placed in a vacuum oven at 40 °C (with a negative pressure of 1 MPa) for 30 min to remove air bubbles. The mixtures were then poured into a mold, and the curing processes were conducted at 80 °C for 1 h, followed by heating to 113 °C for 2 h. Finally, pure E51 and MCSECs (MCSEC-5, MCSEC-10, MCSEC-15, MCSEC-20, and MCSEC-25) were obtained.

### 2.4. Property Testing

A thermogravimetric analysis (TGA) Waters Science and Technology Co., Ltd., USA was used to study the thermal behavior of the composites by using a TGA5500 thermogravimetric analyzer.

A dynamic mechanical analysis (DMA) was used to record the composites’ temperature-dependent viscoelastic properties via a Q800 thermomechanical analyzer (TA Instruments).

A S3400-type scanning electron microscope Hitachi Ltd., Japan.The impact fracture surface morphology of the composites was evaluated using a Charpy impact tester GT-7045-HMH impact testing machine, Gotech Testing Machines Inc., China. In accordance with ISO 179-1:2000, the impact property was examined on at room temperature. The dimension of specimens was 80 mm × 10 mm × 4 mm, with no notches.

WSM-20KN electronic universal testing machine, Intelligent Instrument Equipment Co., Ltd., In Changchun China. The tensile property was investigated on an electronic universal testing machine in accordance with ISO 527:1993. The loading speed was 2 mm/min at room temperature under a load of 20 kN.

The flexural property was manipulated on the electronic universal testing machine in accordance with ISO 178:1993. The loading speed was 2 mm/min at room temperature under a load of 500 N.

In line with SEMI MF 84-1105, the electrical insulation property was tested. The diameters of the cylindrical specimens were 15 mm, and the thicknesses were no more than 3 mm.

The flame resistance was measured using a microcombustion calorimeter on the basis of ASTM D7309-2007a.

## 3. Results and Discussion

### 3.1. Curing Mechanism Analysis

**Figure 1:**
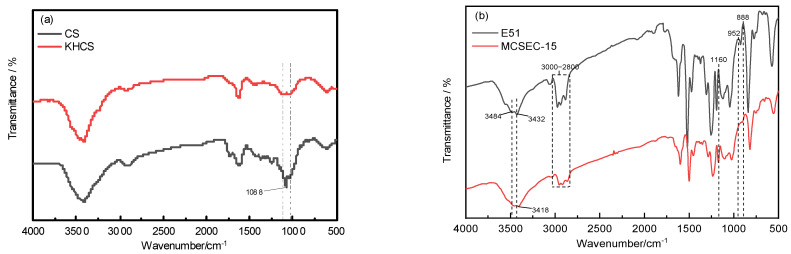
FT-IR spectra (**a**) CS and KHCS; (**b**) curing systems: pure E51 and MCSEC-15.

The structures of CS, KHCS and the curing systems of CSECs were analyzed by FTIR. Figure 1a shows the infrared spectra of CS and KHCS. The stretching vibration peak of Si-O-Si appeared at 1088 cm^−1^ when the CS was modified with KH560, which indicated that KH560 was successfully connected to the CS molecules by covalent bond. Figure 1b presents the FTIR spectra of the pure EP and the composites when the addition amount of KHCS was 10% (MCSEC-10). The stretching vibration peaks of -OH were at 3432 and 3484 cm^−1^, the stretching vibration peaks of –CH_3_ and –CH_2_– were at 3000–2800 cm^−1^, and the wave number at 1160 cm^−1^ was the characteristic peak of C–O–C stretching vibration. The wave numbers at 952 and 888 cm^−1^ corresponded to the stretching vibration peaks of C–O in the epoxy group.

**Scheme 1:**
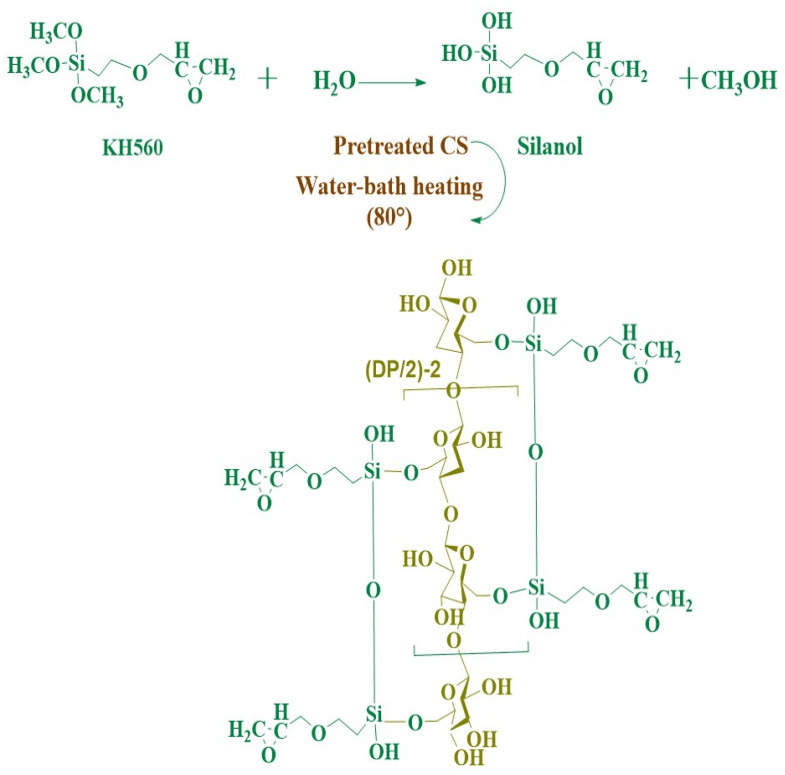
The mechanism of CS modified by KH560.

The mechanism of CS modified with KH560 is shown in Scheme 1. The CS was composed of cellulose, hemicellulose, and lignin. The hemicellulose had dissolved during the pretreating process, and lignin could not react under the reaction condition in this study. Thus, the silane coupling agent was hydrolyzed to silanol functional groups, which increased the number of functional groups and improved the chemical activity. The hydroxyl group of silanol was then reacted with the hydroxyl group at position 6 of cellulose to obtain KHCS [28,29].

Figure 1b illustrates that the infrared characteristic peak corresponding to –OH was at 3484 cm^−1^ for the pure EP, while the characteristic peak moved to 3418 cm^−1^ once the KHCS was added. Li et al. [30] stated that the characteristic peak of –OH moved from a high to a low wave number, which indicated that the number of hydrogen bonds in the –OH network increased. This finding showed that the number of hydrogen bonds in the material structure network was increased due to the addition of KHCS. The reason might be that more hydroxyl groups were contained in the molecules of KHCS, which would form hydrogen bonds with the hydroxyl groups in the molecules of EP.

**Scheme 2:**
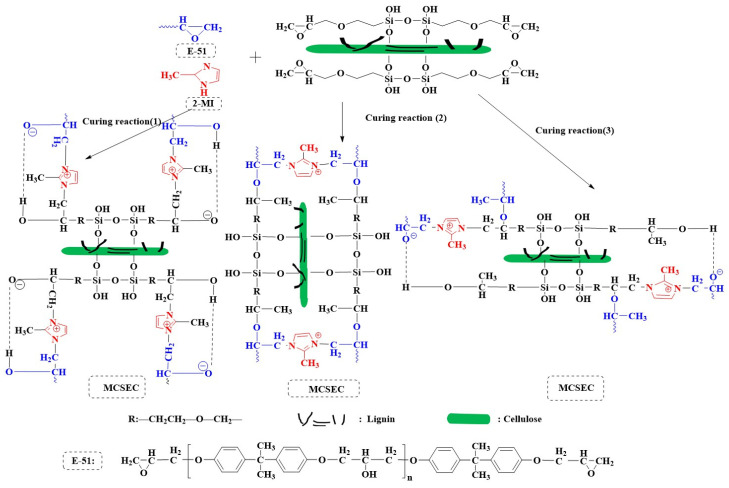
The mechanism of the curing reaction of MCSEC.

The mechanism of this curing reaction is shown in Scheme 2. On the one hand, the secondary amine of 2-MI would present an opening ring reaction with the epoxide group of E51 or KHCS to generate the hydroxyl group, which would exhibit an etherification reaction with the epoxide group to form a cross-linked network structure. On the other hand, the tertiary amine of 2-MI would only present an opening ring reaction with the epoxide group to produce an oxane anion, which could open another epoxy ring to form a cross-linked network structure. The possible reactions are shown in Scheme 2.

### 3.2. Micromorphology Analysis

**Figure 2:**
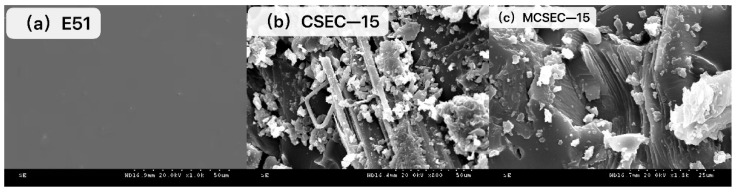
SEM of (**a**) pure E51, (**b**) CSEC-15 and (**c**) MCSEC-15.

Scanning electron microscopy (SEM) is a useful technique that can be used to reflect both mechanical and thermal stability properties [31]. The SEM pictures of the impact fracture surfaces of the composites are shown in Figure 2. Figure 2a depicts that the fracture surface of pure EP was flat and smooth, which was a typical brittle fracture. After CS was added, as shown in Figure 2b, the fracture surface of CSEC appeared to be rough. CS was embedded in EP, but a phase interface between CS and E-51 obviously existed, which indicated that the compatibility between the two phases was poor. Figure 2c shows the SEM picture of the impact fracture surface of MCSEC. Compared with Figure 2b, Figure 2c demonstrates that the surface was rough, and the phase interface between the two phases became blurred, indicating that the compatibility between the modified KHCS and E-51 was improved markedly.

### 3.3. Effects of Particle Sizes and Contents of KHCS on the Mechanical Properties of MCSEC

**Figure 3:**
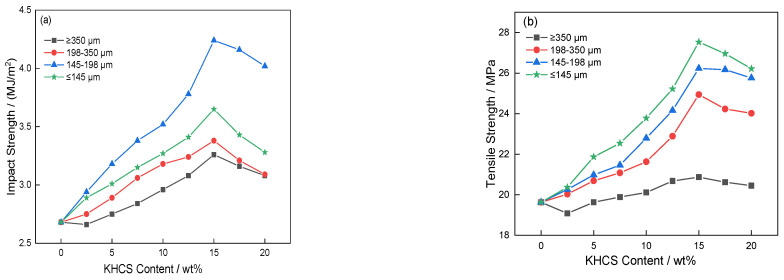

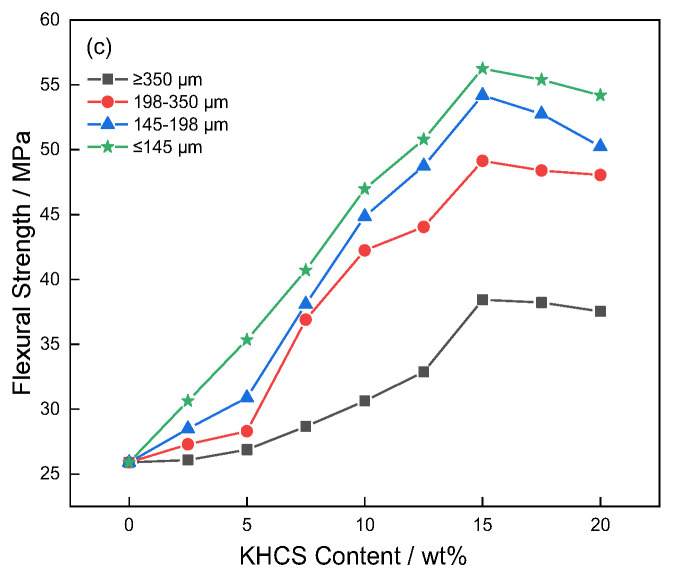
Effects of different particle sizes and contents of KHCS on mechanical properties. (**a**) Impact strength; (**b**) Tensile strength; (**c**) Flexural strength.

The KHCS particles were screened and graded using a screening method, and the particle sizes were graded as ≤145 μm, 145–198 μm, 198–350 μm, ≥350 μm. Then, the KHCS with different mass ratios was mixed with E-51 cured to prepare CSEC specimens. Figure 3 shows the effects of different particle sizes and contents of KHCS on the mechanical properties of the composites.

From Figure 3, the impact strength of the composites first increased and then decreased with the decrease in KHCS particle size. The tensile strength and flexural strength showed an increasing trend. The reason was that the micromorphologies of the straw particles were fibrous with a certain length-diameter ratio, which caused the interaction force and interaction energy between the KHCS and EP matrix to change correspondingly. On the one hand, only if the length-diameter ratio was appropriate would the curing system mix sufficiently that more entanglements between the KHCS and EP were formed. The composites could take advantage of the strength of KHCS to bear the stress, so that the KHCS might deform in the process of stress transfer, which would disperse the stress and prevent the diffusion of cracks, thereby strengthening the composites [32]. On the other hand, the KHCS with large particle sizes would become the stress concentration point of composites, resulting in weakened impact strength [33,34,35].

With the increase in KHCS content, the mechanical properties of the composites first increased and then decreased. When the content of KHCS was higher than 15 wt%, the viscosity of the system became increasingly large, making the system difficult to mix evenly, which might have a certain influence on the mechanical properties of the composites.

Based on the comprehensive properties and the operability of the mixed system, the appropriate particle size of KHCS was 145–198 μm, and the optimum addition amount was 15 wt%.

### 3.4. Dynamic Mechanical Analysis

**Figure 4:**
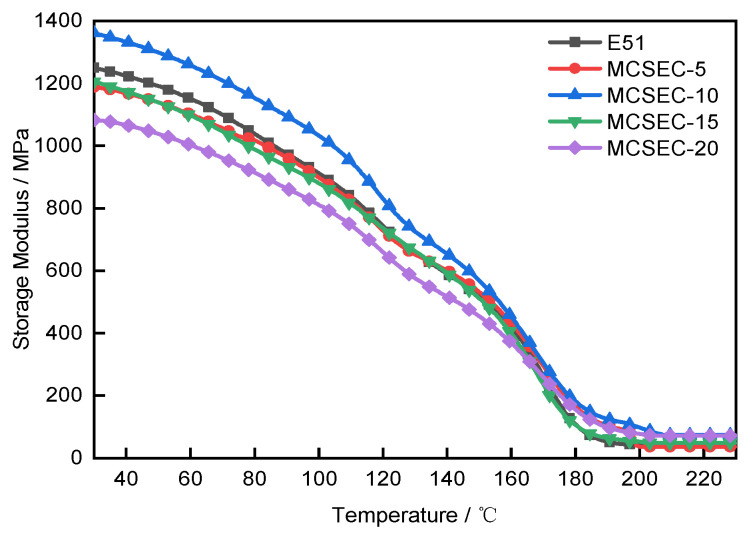
Storage modulus of KHCS/E-51 composites with different contents.

Figure 4 depicts that the initial storage modulus of pure EP was 1250 MPa; that of MCSEC-10 was 1360 MPa, which was the maximum; and that of MCSEC-20 was 1082 MPa, which was the minimum. After different contents of KHCS were added, the initial storage moduli of MCSECs were changed and showed the same trend with the change in temperature. This result was attributed to the fact that the surface of CS short fiber had a large number of active hydroxyl groups, which would bond well with the silane coupling agent and form a stable covalent bond at high temperature. In the EP matrix, the R group of the silane coupling agent penetrated into the molecular chain of the EP and combined with the resin molecular chain by hydrogen bonding [36]. Hence, with the action of two different compatibility groups of the silane coupling agent, the E-51 and straw short fiber were connected through a silicon oxygen bond to form the interface layer of EP–silane coupling agent-straw short fiber, which improved the adhesion between the E-51 and straw. Owing to the large amount of hydroxyl groups in the straw molecules, the curing reaction of this modified system was so complex that a connected network structure would be formed, which was conducive to stress transfer and energy dissipation [37,38]. Therefore, the initial modulus of MCSEC-10 was higher than that of pure EP. In the KHCS short fiber composite with low KHCS content, although the KHCS short fiber could obtain good dispersion, its filling coefficient was not large, and a gap existed between the straw short fiber and the resin. Consequently, it could not give full play to the reinforcement performance of the KHCS short fiber in composites. In the KHCS short fiber composite with high KHCS content, some KHCS in the composite were unevenly distributed, which might damage the overall regular arrangement of EP molecular segments and the uniformity of the distance between cross-linking points. These uneven internal microproperties of MCSEC would lead to concentrated stress in the process of bearing an alternating load, which might cause the deformation resistance and storage modulus of MCSEC to decrease.

**Figure 5:**
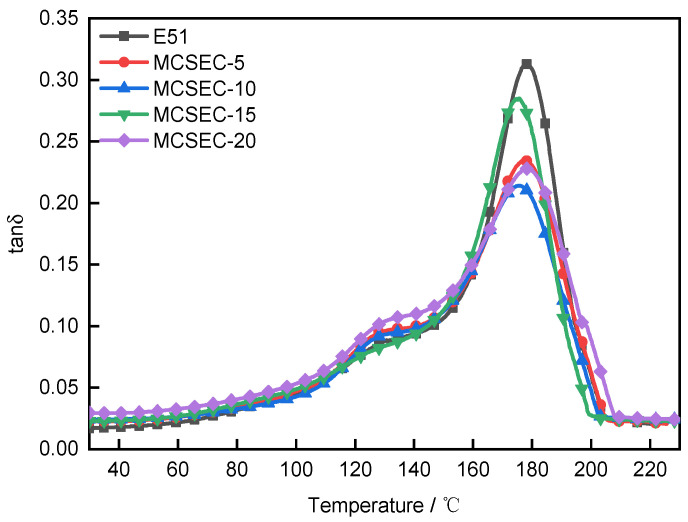
Tan δ of MCSECs with different contents.

Figure 5 shows the peak value of tanδ of the composites in the glass transition temperature region. The change in T_g_ of MCSECs with different addition amounts of KHCS was slightly different from that of pure EP. From Figure 5, the T_g_ of pure EP was 179.4 °C, that of MCSEC-5 was 177.3 °C, that of MCSEC-10 was 175.8 °C, that of MCSEC-15 was 173.4 °C, and that of MCSEC-20 was 178.4 °C. Given that the chain ends of KHCS were more than those of the EP, the movement space of the epoxy molecular segment near the KHCS increased, resulting in an increase in the free volume of MCSEC. This was why the T_g_s of MCSECs were slightly lower than that of pure EP.

### 3.5. Thermogravimetric Analysis

**Figure 6:**
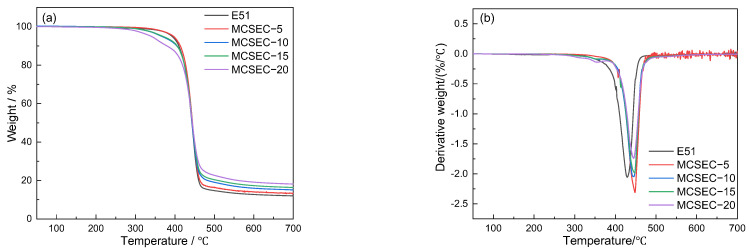
(**a**) TG; (**b**) DTG curves of MCSEC with different contents.

From Figure 6a,b, when the temperature was lower than 400 °C, the weight loss of pure EP and MCSEC was less, and the unreacted small molecules were mainly lost. In the temperature range of 300–400 °C, the movement of molecular chain segments of EP materials intensified, and some groups with poor thermal stability began to decompose. With the increase in temperature, the force between chain segments could not maintain the stability of the whole molecular chain, and fracture occurred between chain segments. After 400 °C, the basic internal structure of the EP and composites was completely destroyed, resulting in a large number of thermal decomposition reactions. At 500 °C, the thermal decomposition was basically completed, and the EP and MCSEC had the same thermal decomposition process. The weight loss of EP was the largest, the maximum thermal weight loss rate temperature was 430.0 °C, and the weight loss rate was about 84.1%. With the increase in KHCS addition, on the one hand, the weight loss rate and maximum thermal weight loss rate of MCSEC gradually decreased, which were lower than those of pure EP, showing simultaneous structural network fracture and carbon formation. On the other hand, the DTG peak of the composite moved to a high temperature, indicating that KHCS improved the thermal stability of the material to a certain extent.

Compared with the initial thermal decomposition temperature and maximum thermal weight loss rate of pure EP, those of MCSEC were reduced when KHCS was added to the EP. KHCS could participate in the curing reaction of EP to form a hydrogen bond, which improved the cross-linking density of the EP matrix. Moreover, the energy of the hydrogen bond was less than that of a covalent bond, which was more prone to bond breaking. When heated, it would decompose first. The sample showed weight loss, and the maximum thermal weight loss rate would also decrease [39,40].

The T_5_, T_10_, T_50_, T_HRI_, and residual carbon at 800 °C of pure EP and its composites are listed in Table 1. The T_5_ and T_10_ of the composites were less than those of pure EP, whereas T_50_ was greater than that of pure EP. To sum up, the thermal conductivity of the composites was better than that of pure EP. When the external heat flowed into the composites, the heat would be preferentially transferred to KHCS and out through the heat conduction path formed between KHCS. The whole heat transfer process caused the temperature of the composite material to rise rapidly. Given that the thermal conductivity of the straw itself would be lower than that of the pure EP, when the composite material heated up, KHCS would accumulate heat and improve the accumulation of heat inside the composite material, reducing the thermal decomposition temperature of the composite material. Therefore, the initial decomposition temperature of the high-content MCSEC was lower than that of the pure EP, and the heat resistance of the EP matrix in MCSEC was higher than that of KHCS. With the increase in content, the heat resistance of the system decreased, resulting in the decrease in T_HRI_ and the slight increase in T_50_. The weight loss of the pure EP and composites was basically stable after 500 °C, and the carbon residue rate of the composites was significantly higher than that of the pure EP at 800 °C. Thus, the addition of KHCS improved the geothermal stability of EP.

### 3.6. Mechanical Properties

**Figure 7:**
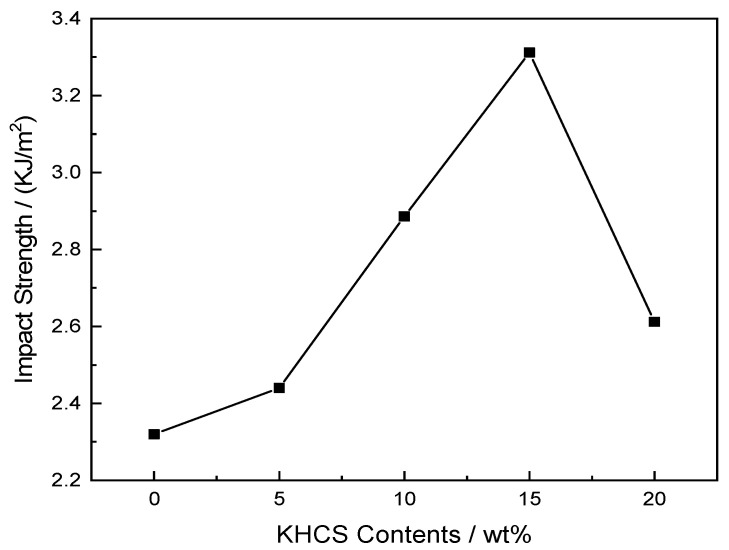
Impact strength curve of the composites.

Figure 7 demonstrates that with the increase in KHCS addition, the impact performance of the composites first increased and then decreased. The impact strength of the pure EP was 2.32 kJ/m^2^. When the addition amount was 15%, the maximum impact strength of the composites was 3.31 kJ/m^2^, which was 1.43 times that of the pure EP. When subjected to external impact stress, microcracks would occur in the material. In the pure EP, once a crack occurred, it expanded rapidly, and the material was destroyed and presented a brittle fracture. In MCSEC, because KHCS had a certain length–diameter ratio and the surface contained functional groups that could react with the EP matrix, the combination between KHCS and EP matrix was enhanced, ran across the front of the crack, played a bridging role, and improved the cracking stress of the EP matrix. It prevented the crack from continuing to expand along the original direction. The crack deflected and extended along the interface between the KHCS and EP matrix. The impact strength of MCSEC increased by consuming more energy through the KHCS pullout and other mechanisms. Therefore, the addition of KHCS could improve the impact toughness of EP. However, with the increase in KHCS content, the number of stress concentration points in the composites increased and the impact strength decreased.

**Figure 8:**
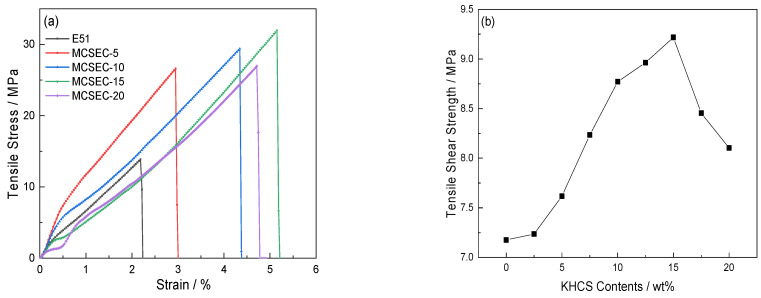

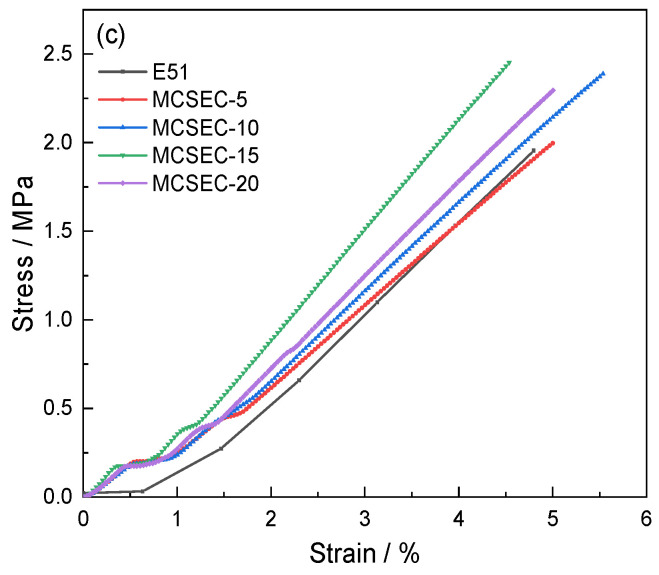
Tensile and bending curves of MCSEC: (**a**) Tensile stress-strain curve; (**b**) Tensile shear strength; (**c**) Flexural stress-strain curve.

According to the analysis of Figure 8, the tensile and bending strength of the composite increased with the addition of KHCS. The number of hydroxyl groups on the straw surface would be reduced after the straw was treated with KH560, which decreased the polarity of the straw surface, improved the hydrophobicity of the straw, and increased the compatibility and bonding strength between the two phases [41]. When the external force acted, the force borne by the EP could be transferred to the straw particles, which improved the tensile and flexural properties of MCSEC. According to the area of stress–strain curve in Figure 8a,c, the addition of KHCS improved the toughness of the composite. The tensile shear strength curve in Figure 8b showed the same trend.

### 3.7. Insulation Properties

**Figure 9:**
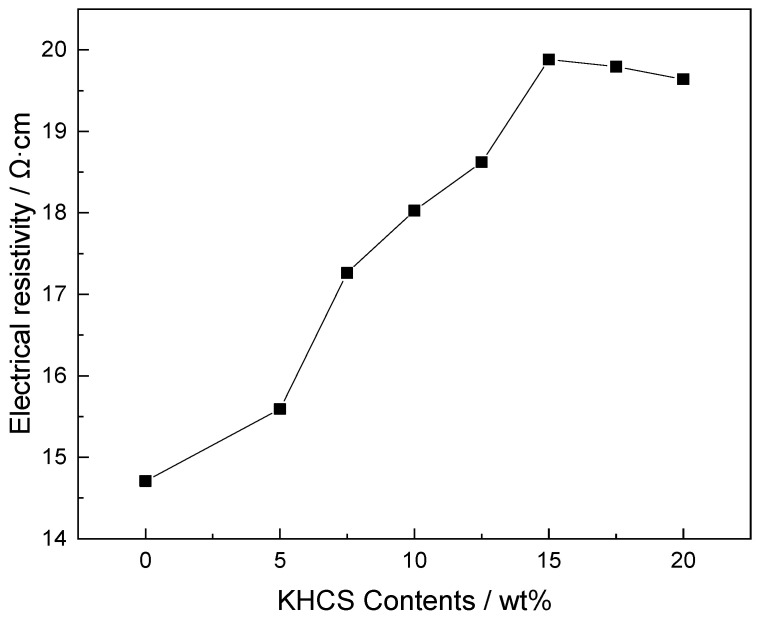
Relationship plot of electrical resistivity and KHCS contents.

The resistivity of the composites after adding different contents of KHCS at room temperature is shown in Figure 9. With the increase in KHCS addition, the resistivity first increased and then decreased. The resistivity of pure EP was 14.71 Ω·cm. After straw particles were added, the resistivity of the system became higher than that of the pure EP. When the addition amount was increased to 15 wt%, the maximum resistivity of the composite was 19.88 Ω·cm, which was 1.35 times that of the pure EP. This result was due to the fact that the electrical insulation performance of CS was stronger than that of pure EP. If the addition amount further increased, the electrical insulation performance of the composite would decline. The reason might be that after the addition amount of KHCS reached a certain level, the KHCS in the composite would agglomerate due to the van Der Waals force, resulting in adsorbed water in the composite and impurities with insulating function attached to the straw surface. Consequently, the current carrying factor in the composite system would increase. The resistivity of the composite decreased by 2 Ω·cm, but it remained at about 19 Ω·cm, which showed that the composite had better electrical insulation performance than EP.

### 3.8. Flame-Retardant Properties

**Figure 10:**
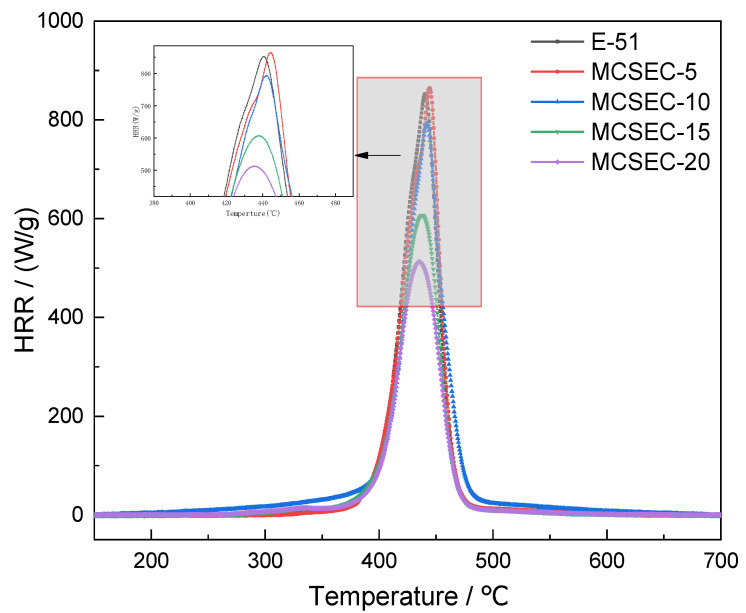
Heat release rate curve of MCSEC.

In the test using a microcombustion calorimeter, the combustion behavior of the sample was close to the real process in the fire scene. The detection and analysis of heat release rate (HRR), peak HRR (p HRR), total heat release (THR), and other data could directly reflect the flame-retardant performance of each system. Specifically, the change in HRR with temperature of systems with different KHCS addition amounts is shown in Figure 10, and the results of p HRR and THR are listed in Table 2. HRR, p HRR, and THR could directly indicate the thermal diffusion degree of the material. The greater the p HRR, the faster the release of heat during the combustion of the material will be, and the easier the combustion of the reaction material will be. From Figure 6, the pure EP and MCSEC-5 released substantial heat at the initial stage of combustion, and the HRR rose sharply. Their maximum p HRR and THR were 864.35, and 852.33 W/g and 34.65 and 34.18 KJ/g. When a certain amount of KHCS was added, the p HRR and THR of the composites decreased significantly. In particular, the p HRR and THR of MCSEC-20 were 512.44 W/g and 25.03 KJ/g, respectively, which were 40.71% and 27.76% lower than those of pure EP. This was because the silanol groups on the surface of KHCS would promote the chemical reaction of the hydroxyl group on the straw surface during the combustion process. At high temperature, the KHCS was decomposed into non-combustible carbon and attached to the surface of the composites to form a stable isolation carbon layer to protect the matrix from further combustion. This finding showed that KHCS had an obvious flame-retardant effect on the EP system.

## 4. Conclusions

Compared with the original CS, the CS that was surface functionalized with a silane coupling agent could better toughen the EP. With the increase in KHCS mass fraction, the mechanical properties of the EP composites basically showed gradual improvement. The best mechanical properties were obtained when the KHCS was 15 wt%, with impact strength, tensile strength, and flexural modulus of 3.31 KJ/m^2^, 21.25 MPa and 63.7 MPa, respectively. A dynamic thermomechanical analysis concluded that for the KH560 surface-treated CS and EP, the van Der Waals forces existing in the matrix were weaker than the entanglement between the EP molecules in the EP matrix. As a result, the Young’s modulus and energy storage modulus of the composites decreased, and the glass transition temperature also decreased compared with that of the pure EP. Thus, the addition of KHCS had a toughening effect on the pure EP.

The silane-coupling-agent-surface-functionalized CS is a better flame retardant and insulating filler. The addition of KHCS will make the composite dehydrate and carbonize during combustion to form a thermal insulation layer and improve the flame-retardant performance of MCSEC. Hence, adding KHCS will enhance the electrical insulation of the composite.

## Figures and Tables

**Table 1:** Thermal stability data of pure epoxy resin and its composites.

Sample	T_5_	T_10_	T_50_	T_HRI_/°C	Residual Carbon/%
E-51	392.72	409.88	443.09	207.24	11.49
MCSEC-5	390.58	402.73	444.38	207.20	14.24
MCSEC-10	370.26	396.04	445.22	203.47	15.71
MCSEC-15	369.53	389.66	445.28	203.34	15.83
MCSEC-20	339.86	379.78	445.89	197.70	17.67

Note: T_5_, T_10_ and T_50_ are the corresponding decomposition temperatures of 5%, 10% and 50% weight loss respectively; T_HRI_ is the heat resistance index.

**Table 2:** Relevant parameters of different systems measured by a cone calorimeter.

System	p HRR (W/g)	THR (KJ/g)
E-51	864.35	34.65
MCSEC-5	852.33	34.18
MCSEC-10	792.95	31.59
MCSEC-15	607.14	28.19
MCSEC-20	512.44	25.03
